# A year in review: brain barriers and brain fluids research in 2022

**DOI:** 10.1186/s12987-023-00429-0

**Published:** 2023-04-21

**Authors:** Richard F. Keep, Hazel C. Jones, Mark G. Hamilton, Lester R. Drewes

**Affiliations:** 1 Department of Neurosurgery, University of Michigan, R5018 BSRB 109 Zina Pitcher Place, Ann Arbor, MI 48109-2200 USA; 2 Bicester, OX26 1UF UK; 3 Department of Clinical Neurosciences, Division of Neurosurgery, University of Calgary, Alberta, Canada; 4 Department of Biomedical Sciences, University of Minnesota Medical School Duluth, Duluth, MN 55812 USA

**Keywords:** Brain endothelium, Blood-brain barrier, Cerebrospinal fluid, choroid plexus, drug delivery, Glymphatics, hydrocephalus, neurovascular unit

## Abstract

This aim of this editorial is to highlight progress made in brain barrier and brain fluid research in 2022. It covers studies on the blood-brain, blood-retina and blood-CSF barriers (choroid plexus and meninges), signaling within the neurovascular unit and elements of the brain fluid systems. It further discusses how brain barriers and brain fluid systems are impacted in CNS diseases, their role in disease progression and progress being made in treating such diseases.

## Introduction

Research related to blood-brain barriers and brain fluids continues to thrive. Here the Editors-in-Chief of *Fluids and Barriers of the CNS* highlight some of the wide range of important articles published in 2022, as well as some controversies. As always, such a review cannot cover the multitude of excellent studies that have been published.

## Elements of the blood-brain barrier and the neurovascular unit

The brain endothelial cells and the surrounding neurovascular unit (NVU) have a crucial role in protecting the brain, forming the blood-brain barrier (BBB), maintaining a homeostatic microenvironment, and in facilitating normal brain function (e.g., neurovascular coupling). The development and maintenance of the BBB, the role of cellular and acellular elements of the NVU in regulating the barrier, and differences from non-barrier areas of the brain (circumventricular organs) have recently been reviewed by Ben-Zvi & Liebner [1]. Crouch et al. [2] have also described the process of angiogenesis and endothelial/mural cell interactions in the 2nd trimester in human brain. It should be noted that the NVU interacts with systemic biology (e.g., the brain-gut axis and the systemic immune system) forming a ‘wider’ NVU [3].

One technique that has had a major impact on our understanding of the cerebrovasculature is the use of single cell transcriptomics. Application of this methodology has provided a mass of publicly available information on the different cell types and sub-types in the NVU (including potential cell specific markers), the existence of arterio-venous zonation, and the impact of disease. Such data is available for humans [4–6] as well as prior and new data in mice (e.g. [7]).

### Brain endothelial cells

Tight junctions (TJ) that link cerebral endothelial cells are an essential component of the BBB and are comprised of transmembrane proteins including claudin-5 and occludin and cytoplasmic plaque proteins including ZO-1. Hashimoto et al. [8] have described a claudin-5 mutation in two patients with alternating hemiplegia with microcephaly that changes this normally barrier forming claudin to an anion-selective channel. Greene et al. [9] have demonstrated the importance of claudin-5 in epilepsy. They report that claudin-5 is reduced and BBB permeability enhanced in patients with drug-resistant epilepsy, and that reducing claudin-5 exacerbates kainate-induced seizures in mice and can induce spontaneous seizures, neuroinflammation and death. Importantly, RepSox, a regulator of claudin-5 could prevent seizures in a mouse epileptic model. Boye et al. [10] have identified the role of Netrin-1-Unc5B signaling in regulating claudin-5 via the Wnt/β-catenin pathway. Endothelial-specific loss of Unc5B reduced claudin-5 expression and disrupted the BBB.

Evidence indicates that beta-site amyloid precursor protein cleaving enzyme 1 (BACE1) has a role in cerebrovascular injury in cerebral amyloid angiopathy and Alzheimer’s disease. Occludin was discovered as a new substrate of BACE1 and leads to cerebral small vessel disease pathogenesis ([11]). Toth et al. [12] have described sortilin as another novel junction regulator. Rats lacking this receptor in brain endothelial cells exhibited a leaky BBB that was associated with a relocalization of the junction proteins ZO-1, VE-cadherin and β-catenin. Loss of sortilin reduced phosphorylation of Akt and increased phosphorylation of Erk1/2.

Major facilitator superfamily domain-containing protein 2 (MFSD2A) plays a major role at the brain endothelium. This dual-function protein transports lysophosphatidylcholines and omega-3 fatty acids and regulates transcytosis. Using cryo-electron microscopy, Martinez-Molledo et al. [13] elucidated the structure of MFSD2A in relation to its transport function. This structural information may allow strategies for development of MFSD2A inhibitors for modulating BBB permeability.

Multiple mechanisms are involved in regulating cerebral blood flow to meet the demands of neural metabolism (neurovascular coupling). In addition, changes in luminal shear stress alter endothelial function as shown by Harraz et al. [14] who identified Piezo 1 channels in endothelial cells that act as mechanosensors and regulate intracellular Ca^2+^ channels using ex vivo retinal preparations and isolated endothelial cells.

### Pericytes and astrocytes at the neurovascular unit (NVU)

Pericytes play an important role at the NVU in controlling brain endothelial function. Thus, Berthiaume et al. [15] have examined the effects of optically ablating pericytes on capillary function in mice and found dilation of capillaries and altered capillary blood flow with no disruption of vascular permeability. However, remodeling of neighboring pericytes was observed and resulted in restored pericyte coverage and capillary tone, a recovery that was slower in aged mice. Ayloo et al. [16] found that vitronectin secreted by pericytes is an important regulator of BBB and the blood-retinal barrier function via interaction with endothelial integrin α5 receptors. The interaction suppressed endothelial transcytosis. Pericytes can produce factors that protect the BBB from hypoxia [17]. Sancho et al. [18] have recently provided evidence that K_ATP_ channels are important regulators of pericyte and brain endothelial cell membrane potentials and that adenosine activates those channels via A_2A_ receptors with important implications for brain blood flow regulation.

Astrocytes are another major regulator of NVU and BBB function. Morales et al. [19] have developed a new way of specifically tracking and ablating perivascular astrocytes using the endogenous gene megalencephalic leukoencephalopathy with subcortical cysts 1 to drive expression of Cre fused to a mutated estrogen ligand-binding domain. They found that loss of perivascular astrocytes led to abnormal location of endothelial junction proteins and microgliosis. This approach should be a useful tool to study the role of perivascular astrocytes. Mills et al. [20] have used two-photon chemical ablation of astrocytes and found that neighboring astrocytes extended their processes to cover denuded areas but this was reduced with aging. Yshii et al. [21] have used a viral gene delivery system (adeno associated virus, AAV) to enhance interleukin-2 production in reactive astrocytes and thereby regulate brain-resident T_reg_ cells. This method was protective in mouse stroke, traumatic brain injury and multiple sclerosis models. Importantly, it did not impact the peripheral immune system.

Aquaporin-4 (AQP4), a water channel, is highly expressed in astrocyte endfeet where it forms large arrays (orthogonal arrays of particles). Zhu et al. [22] have examined the impact of a mutant form of AQP4 (AQP4-A25Q) in mice. They found it greatly reduced the number of arrays without affecting overall mRNA and protein levels. Importantly, they found that the mutant had reduced brain edema, astrocyte endfeet swelling and neurological deficits as well as improved survival after stroke. This emphasizes the functional importance of these physical arrays. Evidence regarding the importance of AQP4 subcellular localization in brain water homeostasis in health and disease is reviewed in Salman et al. [23]. They propose that targeting AQP4 localization may be an alternative strategy to developing AQP4 inhibitors. Evidence on the importance of AQP4 also comes from Mader et al. [24] who found that in utero exposure to maternal anti-AQP4 antibodies affected brain and vascular development and BBB leakiness in male mice.

Astrocytes and brain endothelial cells express connexins important for cell:cell communication. De Bock et al. [25] have shown the importance of connexin 43 in both cell types in BBB disruption following lipopolysaccharide (LPS) administration. Kim et al. [26] have examined the pathways by which inflammatory mediators induce a reactive astrocyte phenotype to then induce BBB disruption. Tumor necrosis factor-α induced a reactive phenotype via STAT3 and SERPINA3 upregulation (encoding alpha 1-antichymotrypsin). Apoliprotein E (ApoE) is produced in the brain by astrocytes and microglia as well as systemically by the liver. In humans, the ApoE4 allele is associated with increased risk of Alzheimer’s disease and a leaky BBB. Jackson et al. [27] have found that mice expressing human APOE4 have a leaky BBB, including impaired TJs and reduced astrocyte end-foot coverage. Removing astrocyte-produced ApoE4 reduced that phenotype indicating an important role of that cell type. Barisano et al. [28] have used a multi-omics approach to examine the effects of knocking-in human APOE4 into mice particularly focusing on brain endothelial dysfunction and pericyte damage. While APOE4 is abundant in brain, it is also present in the periphery. Liu et al. [29] have found that peripheral APOE4 has adverse effects on the cerebrovasculature and cognition in mice. This suggests that targeting peripheral APOE4 may be a therapeutic strategy for Alzheimer’s disease.

Gerrits et al. [30] have used single-nucleus RNA sequencing to examine changes in the NVU in patients with frontotemporal dementia with heterozygous mutations in the progranulin gene. They identified disease-associated endothelial and astrocyte subtypes and a loss of pericytes.

While most studies have focused on how components of the NVU impact brain endothelial cell function, there is important communication in the opposite direction. For example, Li et al. [31] found brain endothelial cells release microvesicles containing Ascl1 during stroke-like conditions in vitro that convert astrocytes into neural progenitors. Endothelial specific overexpression of Ascl1 improved behavioral outcomes in mice after stroke. Endothelial platelet-derived growth factor B (PDGFB) plays a crucial role in brain angiogenesis by recruiting pericytes. Vazquez-Liebanas et al. [32] have examined the effects of loss of PDGFB in adult mice after angiogenesis and found that PDGFB loss led to a gradual reduction in endothelial pericyte coverage and increased BBB permeability. Lee et al. [33] have also found that endothelial-derived lactate is a substrate fueling pericyte metabolism.

One important function of signaling at the NVU is in neurovascular development. This requires co-ordination so that vascular development meets the metabolic needs of neural cells. Rattner et al. [34] describe current knowledge of the signaling pathways involved.

### Oligodendrocytes

While attention has focused on the NVU in grey matter, there is also an analogous structure in white matter termed the oligovascular unit (OVU). The OVU contains brain endothelial cells, oligodendrocytes and oligodendrocyte progenitor cells (amongst other components). Xiao et al. [35] have found that IL-17/CXCL5 signaling in the OVU plays an important role in white matter injury in humans and mice.

### Microglia

In contrast to the role of astrocytes and pericytes at the NVU, the role of microglia has received relatively little attention. Knopp et al. [36] has reviewed evidence on the connection between microglia and the cerebral capillaries (~ 30% of microglia are connected to a capillary) and the effects of disease states on microglia-endothelial communication. Very recently, Csaszar et al. [37] have described the importance of microglia in neurovascular coupling under normal and hypoperfusion conditions, roles mediated by purinergic signaling.

### Fibroblasts

One often neglected CNS cell type are the fibroblasts. They are present in the perivascular space, the choroid plexus stroma and the meninges. They also play a key role in scar formation after brain injury. The role of these cells has recently been reviewed [38].

### Extracellular matrix

The extracellular matrix is an important component of the NVU. Halder et al. [39] have recently reviewed how genetically manipulating laminins and their integrin receptors impact BBB development/maturation and their role in maintaining BBB integrity. Nirwane and Yao [40] have also recently reviewed the cell specific expression of different laminins within the NVU and their function. De et al. [41] have found that β8-integrin activates extracellular matrix adhesion to promote cerebrovascular development. β8-integrin is essential for transforming growth factor β-dependent gene expression in the endothelium.

The glycocalyx on the luminal surface of the cerebral endothelium also has many important functions. Zhu et al. [42] have found that glycocalyx destruction, such as in stroke, is associated with increased transcellular, caveolin-1 mediated, transport at the BBB.

## Elements of the blood-CSF barrier and the CSF and glymphatic systems

### Choroid plexus

The choroid plexus is thought by most to be the primary site of CSF secretion with a lesser contribution from the neurovasculature. In terms of CSF secretion, there has been particular focus on the role of the sodium/potassium/chloride cotransporter, NKCC1 [43–46], and transient receptor potential vanilloid 4 (TRPV4) channels [44, 47] at the choroid plexus epithelium. Such research may lead to a clinical method for reducing CSF production. There is still debate about the mechanism(s) by which fluid is transported across the choroid plexus epithelium. That is reviewed by MacAulay et al. [48] with a particular focus on the hypothesis that certain transporters (e.g., NKCC1) may serve as a water channel as well as transport solutes. There has been great interest in how fluid movement in the brain varies diurnally particularly in relation to removal of waste or toxic metabolites (e.g., via the glymphatic system) and it should be noted that the choroid plexus has a circadian rhythm that may impact CSF homeostasis and flow (reviewed in [49]).

The choroid plexus is also increasingly understood to play a major role in neuroinflammation. For instance, Alzheimer’s disease causes marked changes in choroid plexus myeloid cells [50]. The NLR family pyrin domain containing 3 (NLRP3) inflammasome is involved in CP hypersecretion after intraventricular hemorrhage [46]. The choroid plexus is also a target for reducing T cell infiltration in experimental autoimmune encephalitis [51] and there are very early inflammatory changes at the choroid plexus after intracerebral hemorrhage [52].

As with the cerebral endothelium, choroid plexus development is regulated by Wnt/b-catenin signaling. Parichha et al. [53] have examined the effects of disrupting such signaling in mice and organoids from human embryonic stem cells. Both loss and gain of function of β-catenin disrupted choroid plexus epithelium development indicating the importance of tight regulation of Wnt/β-catenin signaling for correct choroid plexus development.

There have been an increasing number of reports using MRI to examine changes in the choroid plexus in different human disease states. That includes increases in human choroid plexus size or volume in multiple sclerosis (including pre-symptomatic) [54–56], Alzheimer’s disease [57], depression [58] and obesity [59]. Such changes may reflect neuroinflammatory events at the choroid plexus as evidenced by positron emission tomography [56, 58]. MRI has also been used to examine changes in choroid plexus function in patients including perfusion [60], water movement [61] and contrast permeability [57].

Understanding the roles of the choroid plexus would be greatly assisted by developing methods to specifically target choroid plexus epithelial cells to induce loss or gain of function. Jang & Lehtinen [62] have recently reviewed progress in that area. It would also be assisted by developing new in vitro choroid plexus epithelium models. Hulme et al. [47] have described a human choroid plexus papilloma (HIBCPP) cell line that had morphology similar to the epithelium in vivo, intermediate transepithelial electrical resistance and correct ion transporter location. Very importantly, these cells secreted fluid when transient receptor potential vanilloid 4 (TRPV4) channels were activated. In contrast, the same group found the porcine choroid plexus-Riems (PCP-R) cell line, while having a high electrical resistance, had incorrect ion transporter location [63].

### Ciliated ependyma

Ependymal cell motile cilia produce CSF flow patterns on the cell surface which may provide a communication route between different periventricular regions. Faubel et al. [64] have been examining the CDKL5 deficiency disorder (CDD), the most common cause of infant epilepsy. CDKL5 regulates cilia length and function, and Faubel et al. found longer cilia, abnormal cilia motion and altered CSF flow patterns in the ventral 3rd ventricle in Cdkl5 knockout mice. Mutations in another epilepsy-related gene, Yes1, produced similar alterations in cilia function and CSF flow patterns. Animals with these altered flow patterns had increased risk of anesthesia-induced seizures and the authors suggest that altered motile cilia function has an essential role in CDD related seizures and that cilia may be a therapeutic target. Changes in the ependymal/cilia function are hypothesized to be crucial in hydrocephalus and recent studies are described in the ‘Hydrocephalus’ section below.

### Cerebrospinal fluid

The importance of CSF composition has recently been stressed by results from Iram et al. [65]. They found that infusing CSF from young mice enhanced oligodendrogenesis and memory in aged mice. Effects on oligodendrocyte progenitor cells were mediated by serum response factor (a transcription factor) which was activated by fibroblast growth factor 17 in the CSF of young mice.

There continue to be many studies examining CSF for biomarkers of disease. This has been a particular focus in Alzheimer’s disease where studies have examined the correlation between CSF biomarkers and disease pathology [66–68], examining potential biomarkers that are upregulated before the onset of cognitive impairment [69] and distinguishing between different types of dementia [70, 71]. Apart from β-amyloid (Aβ) and tau proteins, there has been particular interest in microglia-derived sTREM2 (soluble triggering receptor expressed on myeloid cells 2) [72–74]. One biomarker-related question is how well CSF and plasma levels correlate? Shahim et al. [75] examined that question in professional athletes with repetitive head injury. They found that plasma levels of T-tau, GFAP, Aβ40, and Aβ42 do not correlate with CSF levels and concluded that the plasma levels of those biomarkers are not informative for diagnosing the late effects of repetitive brain injury.

Eninger et al. [76] have used non-directed liquid chromatography/mass spectrometry to examine the age-related CSF proteome in mouse models of β-amyloidosis and α-synucleinopathy. They found most of the CSF proteome changes were linked to microglia and astrocytes, identifying a panel of 20 glial-derived proteins. They found a good correlation between those proteins and disease-associated glial genes previously identified by single-cell transcriptomics. Lobanova et al. [77] have shown that it is possible to identify protein aggregates in CSF and plasma using optical single-molecule imaging and high-resolution atomic force microscopy. They compared samples from control and Parkinson’s disease patients and found larger aggregates with a different composition in Parkinson’s disease (50% α-synuclein and 50% Aβ versus 30%:70% in controls).

Cerebrospinal fluid is also an alternative to a brain biopsy for examining brain tumor DNA and RNA [78, 79]. The advantages (and disadvantages) of this approach and comparisons to using blood circulating tumor DNA have recently been reviewed by Tivey et al. [80].

A variety of techniques are available to profile immune cell changes in the CSF in disease states, including cell transcriptomics. Piehl et al. [81] have used RNA analysis to examine CSF changes during normal aging and cognitive impairment. They found that monocytes signal to clonal CD8 + T cells via CXCL16-CXCR6 in patients with cognitive impairment. Yazdani et al. [82] have used the same technique to examine the CSF of patients with amyotrophic lateral sclerosis (ALS) and described clonally expanded CD4^+^ and CD8^+^ T cells with characteristic patterns of gene expression.

### Meninges

The functions of the arachnoid membrane (blood-arachnoid barrier) have been relatively understudied. However, recent studies have indicated that the arachnoid membrane highly expresses many solute transporters regulating CSF composition. For example, Takeuchi et al. [83] have used quantitative targeted absolute proteomics to compare the expression of a variety of transporters at the blood-arachnoid barrier and the blood-spinal cord barrier. Some organic anionic and cationic drug transporters were only expressed in the former. Sangha et al. [84] have found two important folate transporters, the reduced folate-carrier and the proton-coupled folate transporter, are present at the blood-arachnoid barrier.

### CSF circulation and outflow

Two recent reviews have dealt with methods of measuring CSF secretion rate [85] and using different imaging techniques to examine CSF flow [86]. There is debate about the relative importance of different CSF outflow pathways (e.g., across cribriform plate, along cranial nerves, via dural lymphatics or arachnoid villi). Decker et al. [87] used MRI to track CSF outflow in young and old mice and found that it was predominantly via the cribriform plate to the nasopharyngeal lymphatics and that CSF turnover was reduced in aged mice. Albayram et al. [88] have also used MRI to track brain lymphatic networks in human. Using lymph albumin as a marker, they identified lymphatic structures along the dural venous sinuses and along cranial nerves in the dorsal and ventral regions, respectively. They also noted cervical lymph node atrophy and thickening of lymphatics channels with aging.

As well as being involved in CSF drainage, the meningeal lymphatic endothelial cells have phagocytic activity that can take up proteins, polysaccharides and virus particles [89]. Li et al. [90] have also found that several neurotropic viruses drain from the brain via the meningeal lymphatics to the cervical lymph nodes and blocking such drainage increased neurological damage and mortality in virus-infected mice.

There also continues to be great interest in the newly described skull/dural channels linking the subarachnoid space to the skull bone marrow. Pulous et al. [91] have reported that in mice CSF tracers can exit the subarachnoid space and migrate to the skull bone marrow. In meningitis, they found that bacteria use this route to invade the skull hematopoietic niche. Similarly, Mazzitelli et al. [92] described that after a spinal cord injury in mice CSF migrates to the bone marrow hematopoietic niche promoting myelopoiesis and migration of myeloid cells into meninges.

The relationship between cardiac cycle and CSF flow is poorly understood. Yang et al. [93] have used functional MRI (fMRI) to examine the temporal relationship between hemodynamic changes (tissue blood volume) and CSF flow.

### Glymphatics

The glymphatic system continues to generate much interest and some controversy (see [94–98] for recent reviews). Impairments in the rate of perivascular penetrance of CSF (or CSF tracers) into brain has been reported in numerous neurological conditions including multiple sclerosis [99], small vessel disease [100], chronic poor sleep [101], and raised intracranial pressure [102]. Also, movement of CSF into brain has been proposed to be a significant source of early brain edema after anoxia [103].

Drieu et al. [104] have recently identified a sub-population of perivascular macrophages closely associated with the brain arterial tree that regulate arterial motion and associated CSF flow. Loss of the perivascular macrophages impaired access of CSF to the perivascular space. Mestre et al. [105] have examined pial structure in mice, how it may influence perivascular fluid flow (and CSF filtration), and how pial structure is altered with aging and Alzheimer’s disease.

## Genetics and the blood-brain barriers

In 2022, FBCNS published a series of articles in a thematic series entitled, ‘Genetic Disorders and Genetic Manipulation at the Blood-brain Barriers’ (Genetic disorders and genetic manipulation at the blood-brain barriers (biomedcentral.com)). Studies on patients with genetic disorders, and the use of animals with genetic manipulation have provided insight into the role of particular proteins in blood-brain, blood-retina and blood-CSF barrier function in health and disease. Hopefully, such information will provide ways to treat genetic disorders and manipulate barrier function to treat neurological disease.

An example of such research is the use of induced pluripotent stem cells (iPSCs) from patients with genetic disorders to produce brain microvascular endothelial cell (BMEC)-like cells to examine changes in BBB function in vitro. For example, Linville et al. [106] have used such cells to examine the impact of Huntington’s disease mutations on the function and properties of the BBB.

Gene delivery is one potential method for treating genetic disorders. Sundaram et al. [107] have found that using an AAV directed to deliver the monocarboxylic acid transporter 8 (AAV-BBR1-Mct8) to the brain endothelium of mouse models of Allan-Herndon-Dudley syndrome could increase brain tri-iodothyronine (T3) levels and ameliorate morphological and behavioral deficits.

Transgenic mice have proven a valuable tool for determining the role of specific proteins at the blood-brain, blood-retina and blood-CSF barriers and the NVU. For example, Goncalves & Antonetti [108] have recently reviewed the insights gained from such animals on BBB and blood-retina barriers and their regulation. Halder et al. [39] have also recently described the impact of genetically manipulating laminins and integrins.

Apart from DNA mutations, changes in gene (and protein) expression can occur at multiple levels, The role of epigenetics at the blood-brain barriers is relatively understudied. There is recent evidence that the demethylation factor Tet methylcytosine dioxygenase 2 (TET2) regulates the tight junction protein ZO-1 [109] and p-glycoprotein (ABCB1) [110] and that differences in methylation of the claudin-5 gene are associated with the trajectory of cognitive decline in patients [111].

Another level of regulation is by non-coding RNAs (including microRNAs, long non-coding RNAs and circular RNAs. Sun et al. [112] have reviewed the roles of such RNAs at the BBB in different neurological disorders. One mechanism by which extracellular vesicles shed by one cell can affect other cells is by encapsulating micro RNAs. Brain endothelial cell-derived vesicles are an example of this mechanism [113].

## Neurological disorders

BBB/NVU function and brain fluid dynamics are altered in many neurological disorders. Indeed, there is increasing evidence that targeting such dysfunction can ameliorate the effects of several diseases. This section highlights progress in understanding such changes in select diseases.

### SARSCoV-2/COVID-19

There continue to be extensive studies examining the neurological and neuropsychiatric consequences of SARS-CoV-2 (COVID-19) infection, including long-term effects (long COVID). Potential underlying mechanisms have recently been reviewed [114]. In patients dying with infection, Agrawal et al. [115] found evidence of vascular pathology and microglial activation in the majority (80%) of subjects. In the elderly, pathological changes were superimposed on pre-existing brain disease, making that population more at risk of neurological sequelae. Etter et al. [116] found that COVID-19 patients with severe neurological effects had BBB impairment, markers of microglia activation and a polyclonal B cell response. Similarly, in CVID-19 patients who died with minimal respiratory involvement, Lee et al. [117] reported multifocal vascular damage with BBB disruption and activation of endothelial cells and the classical complement cascade. Jarius et al. [118] found evidence of blood-CSF barrier disruption in patients with neurological sequelae that was still present 30 days post-infection.

Understanding the neurological effects of COVID-19 may be aided by studies on non-human primates. In such a model, Rutkai et al. [119] found evidence that COVID-19 induces hypoxia-ischemia injury with brain microhemorrhages, hypoxia, neuroinflammation and neurodegeneration. Virus was only detected sparsely in brain endothelial cells and did not correlate with brain injury severity. It should be noted, however, that Altmayer et al. [120] found increased plasma endothelial biomarkers in patients with COVID-19-associated encephalitis, although whether those effects are linked to direct endothelium infection or secondary effects is uncertain. Krasemann et al. [121] found SARS-CoV-2 can infect human iPSC-derived BMEC-like cells in vitro.

### Hydrocephalus

Hydrocephalus is defined as enlarged cerebral ventricles with or without raised intracranial pressure and is a disorder of brain fluid circulation. Several major causes exist for hydrocephalus: congenital, usually due to genetic or developmental defects, brain hemorrhage, brain trauma or anoxia, and infections. Additionally, normal pressure hydrocephalus of the elderly has uncertain causes. The major causes of hydrocephalus may vary globally. Aukrust et al. [122] recently analyzed studies from Africa and found that post-infectious hydrocephalus is the single most common cause (28% of cases). The prevalence of hydrocephalus is also strongly age dependent. Thus, Isaacs et al. [123] reported an 8-fold decline between pediatric (perinatal to 18 years old) and adult (19 to 64) populations, but a 17-fold increase from adult to the elderly (65 years and above).

#### Genetic factors

Genetic factors frequently involve altered ependymal cell and cilia function as an underlying cause of hydrocephalus. For example, Han et al. [124] have examined the effects of the deletion of the RNA binding protein Hu antigen R (HuR) in mice. HuR deletion resulted in impaired ependymal cell development, defective motile ciliogenesis and hydrocephalus. They found that HuR binds to mRNA transcripts related to ciliogenesis, including cilia and flagella-associated protein 52. Harkins et al. [125] have examined the effects of loss of nuclear factor one X (NFIX) in mice which is a transcription factor involved in normal ependymal development and the constitutive loss of NFIX results in hydrocephalus. They found loss of ependymal NFIX in adult brains causes enlarged ventricles, abnormal localization of adhesion molecules and shedding of ependymal cells. *Prh* mice have a point mutation in the Ccdc39 gene causing ciliary dyskinesia and progressive hydrocephalus. The mutation also causes periventricular white matter injury and neuroinflammation. Iwasawa et al. [126] found that an anti-inflammatory agent, Bindarit, improved cortical development and reduced white matter injury in these mice while having only a mild effect on ventricular volume.

It should be noted that the hypothesis that ependymal motile cilia dysfunction causes hydrocephalus by altering CSF flow has been questioned by Duy et al. [127]. They suggest that some of the mutations that induce motile cilia dysfunction and induce hydrocephalus may also have independent effects on neurodevelopment and non-CNS effects that may result in the ventricular enlargement. They also note that motile ciliopathies rarely cause hydrocephalus in humans (in contrast to mice). In line with this, the same group examined genetic mutations linked to congenital hydrocephalus [128]. They found convergence of risk genes in embryonic neuroepithelial stem cells. TRIM71 harbored the most de novo mutations and it is specifically expressed in neuroepithelial cells. In mice, neuroepithelial-specific Trim71 deletion or mutation induced hydrocephalus. This may be due to premature neuroepithelial cell differentiation, reduced neurogenesis and cortical hypoplasia. The latter may increase cortical compliance and induce secondary ventricular enlargement.

#### Intraventricular hemorrhage

One major cause of hydrocephalus is intraventricular hemorrhage (IVH). In premature infants IVH is due to germinal matrix hemorrhage and survivors can have significant developmental delay [129]. Blood products in the ventricles are suspected to be responsible for the dilatation and Miller et al. [129] have shown that hemoglobin injected into the ventricles of neonatal rats can induce hydrocephalus. Neuroinflammation is also involved in IVH-induced hydrocephalus (reviewed in [130]). Inflammatory markers in the CSF are associated with hypersecretion of CSF via hyperactivity of choroid plexus transporters, raising the potential for future pharmaceutical treatment [43]. Methods for mitigation of the effects of IVH are being explored and Vinukonda and La Gamma [131] used stem cell lines to improve outcome in animal models. In adults, the CLEAR III trial provided evidence that intraventricular fibrinolytics improve mortality after IVH, but there was no significant difference in functional outcome [132]. Kuramatsu et al. [133] have now performed a meta-analysis of studies using intraventricular fibrinolytics for intracerebral hemorrhage-induced IVH in adults and found significant improvement in functional outcome and that this was linked to early (< 48 h) treatment. This may inform future trials.

#### Normal pressure hydrocephalus

Normal pressure hydrocephalus (NPH) is a condition occurring in the elderly showing dilated ventricles, together with gait, urinary and cognitive dysfunctions, but without consistent raised CSF pressure. The problem of identifying patients that would benefit from shunt treatment remains ongoing and CSF biomarkers are being investigated extensively. Among a series of biomarkers tested, phosphorylated-tau and total-tau have been found to be significantly increased in lumbar CSF of shunt non-responsive patients using a meta-analysis [134], although earlier studies have refuted this and more sophisticated analytical methods may be helpful in the future. Neurofilament light (Nfl), a protein of axonal degeneration, has also been suggested as a biomarker for NPH [135]. Alzheimer’s disease (AD) and NPH often co-exist but after shunt, patients with AD have a worse outcome cognitively [136].

Magnetic imaging studies have shown that NPH is associated with a decrease in grey matter in some brain areas and increase in others and in particular the cerebellum was adversely affected [137]. MRI analysis (cine-PC) has been used to evaluate CSF and brain recovery, including tissue stiffness, over 15 months after shunting [138]. Intracranial compliance after shunting is slow to recover and shows oscillatory changes over 12 months and does not follow measured changes in intracranial pressure or CSF volume [139]. Such information could be used in the future for post shunt patient management. EEG analysis together with normalized power variance as a measure of cortical activity, is also being explored to distinguish shunt responsive NPH patients [140]. The underlying cause for NPH is elusive although dysfunction of the glymphatic system may be implicated (reviewed in [141]).

#### Idiopathic intracranial hypertension

Idiopathic intracranial hypertension (IIH) is a condition of raised intracranial pressure without dilatation of the ventricles most frequently found in obese females. The most common symptoms are severe headache and papilledema which can lead to visual loss. Ultrasound scans of the optic nerve are a reliable diagnostic test [142]. Magnetic resonance elastography has detected changes in the pituitary position and increase in brain stiffness in patients with IIH [143]. A general metabolic dysregulation has been detected in IIH patients [144] and Westgate et al. [145] found changes in systemic glucocorticoid metabolism with an increase in11β-hydroxysteroid dehydrogenase (11β-HSD1) and 5α-reductase activity which decreased after weight loss and a fall in ICP. Changes in the pituitary and pineal glands have also been implicated [146]. Treatment for IIH is designed to reduce CSF pressure and frequently involves CSF shunting but this can result in over-drainage and has a high revision rate (reviewed in [147]).

Fenestration of the optic nerve to release pressure has been tested as a treatment but with limited effect [148]. Causes for this condition are elusive and dural venous sinus stenosis treated by stenting has received much attention (reviewed in [149]). Diet-induced obesity leads to a similar condition in rats providing a reliable animal model [150].

### Stroke

Neuroinflammation plays a central role in brain injury and brain repair following stroke (reviewed in [151]). One important element of the inflammatory response is leukocyte influx across the BBB. Multiple approaches have been used to try and reduce such extravasation. Recently, Arias et al. [152] found that a PECAM-1 neutralizing antibody reduces leukocyte influx and affects their spatial distribution within brain. BBB disruption occurs after stroke and it contributes to neuroinflammation and edema formation. Thus, for example, Ng et al. [153] found that midline shift (a measure of brain edema on imaging) correlates with the degree of BBB disruption as well as poor patient outcome after large vessel ischemic stroke.

Sphingosine-1-phosphate (S1P) regulates BBB permeability with differential effects via the S1P receptors 1 and 2. Hansen et al. [154] have found that the S1P receptor 4 is also present at the cerebral endothelium (apical membrane) in multiple species. Using KO mice, siRNA as well as antagonists and agonists, they found this receptor protects against BBB disruption and that receptor expression is reduced after stroke. Recent evidence points to osteopontin having a role in stroke [155]. Osteopontin disrupted the BBB in vitro and a neutralizing antibody reduced glial activation, BBB disruption and brain edema as well as improving neurological outcome and survival after stroke in mice.

Cerebral small vessel disease is a major cause of stroke, cognitive decline and dementia. Wardlaw et al. [156] have reviewed human and preclinical data indicating how altered endothelial cell dysfunction in cerebral small vessel disease impacts cell:cell interactions in the NVU and leads to brain injury. Mishra et al. [157] have used gene mapping to identify mutations in TRIM47 as being important in cerebral small vessel disease pathology. Using a novel rat KO line, Quick et al. [158] have provided evidence that loss of the phospholipase flippase, ATP11B, causes endothelial dysfunction and white matter injury similar to human cerebral small vessel disease.

Wnt/β-catenin signaling is an instrumental pathway for inducing barrier properties of brain endothelial cells. Ji et al. [159] tested lithium, a known activator of Wnt signaling, and found a protective effect against ischemic stroke and reperfusion injury. Therefore, agents that activate Wnt signaling may have therapeutic value following cerebral vascular insults. This concept was further developed by Martin et al. [160] who report an elegant study in which Wnt7a ligands were genetically engineered into BBB-specific Wnt activators that mitigated ischemic stroke infarction and glioblastoma expansion. They demonstrated that the signaling specificity of Wnt ligands is adjustable and targetable, thus, defining the normalizing of the BBB/NVU as a new modality to treat CNS disorders.

Ischemic and hemorrhagic stroke induce different injury mechanisms. Solar et al. [161] have recently written a very extensive review on the effects of subarachnoid hemorrhage on the BBB and NVU.

### Cerebral cavernous malformations (CCMs) and arteriovenous malformations (AVMs)

Inherited CCMs are due to loss-of-function mutations in three genes, CCM1, CCM2 and CCM3. Animal and cellular models have been generated to examine how these loss of function mutations lead to CCMs [162, 163]. Such models allow examination of potential therapeutics and investigation of factors that may exacerbate CCM lesion development. Thus, McCurdy et al. [164] have found that a β1-integrin monoclonal antibody reduces CCM1 lesion development in mice, and Lai et al. [165] have examined the role of neuroinflammatory astrocytes in CCM3 lesion development. Interestingly, Fang et al. [166] found that loss of the NOGOB receptor in brain endothelial cells results in CCM-like lesions with dilated vessels, BBB hyperpermeability and cerebral hemorrhage. This was linked to an impairment in histone acetylation-mediated CCM1/2 expression.

To understand the abnormal vasculature in AVMs, Winkler et al. [6] compared single cell transcriptomics of the normal human cerebrovasculature and AVMs. They particularly identified pathological endothelial transformations and vascularly derived inflammation.

### Aging and dementias

Normal aging in patients impacts the BBB resulting in small leaks detectable on dynamic contrast-enhanced magnetic resonance imaging [167]. The effects of aging can be exacerbated in patients with Alzheimer’s disease and other dementia’s such as frontotemporal dementia [30, 167, 168]. Yang et al. [5] have developed a ‘vessel isolation and nuclei extraction for sequencing’ technique to examine vascular and perivascular cell transcriptomes (and arteriovenous zonation) in brains from individuals with Alzheimer’s disease and those without cognitive impairment. They found evidence that there is selective pericyte vulnerability and blood flow dysregulation in Alzheimer’s disease brains; that two thirds of the top genes associated with risk of Alzheimer’s disease are expressed in the vasculature; and that those genes are linked to endothelial protein transport, adaptive immunity and extracellular matrix pathways. Consequently, activation of the Wnt/β-catenin pathway may be one way of ameliorating the vascular effects of Alzheimer’s disease [169].

Two recent studies have examined how the vascular transcriptome is changed in mice with cerebral amyloid angiopathy (CAA; Swedish mutation) during aging [170, 171]. Both studies highlight the inflammatory changes, alterations in endothelial biology and the impact of age on the transcriptome. There are always concerns over how well mouse genetic models replicate human CAA (and Alzheimer’s disease in general). Tachida et al. [172] have created a new mouse model with expanded CAA pathology by crossing mice expressing human amyloid precursor protein (APP) in endothelial cells with knock-in mice expressing a construct containing a humanized Aβ region along with two pathogenic mutations (Swedish and Iberian). The relevance of this model remains to be evaluated.

### Neuroinflammation, multiple sclerosis (MS) and amyotrophic lateral sclerosis (ALS)

Proulx and Engelhardt [173] recently reviewed evidence on immune privilege and immune surveillance in the CNS, with the latter being primarily within the subarachnoid and perivascular spaces. Activation of the immune system has a detrimental effect in a variety of neurological disorders. For example, Frieser et al. [174] identified an important role of resident CD8^+^ T cells in autoimmune encephalitis and Vincenti et al. [175] found that after clearance of an infection with attenuated lymphocytic choriomeningitis virus reactivation of tissue-resident CD8^+^ T cells may cause immunopathology, but cooperation of CD4^+^ T cells is required.

Neuroinflammation is a crucial component of MS with autoreactive lymphocytes attacking CNS myelin. For example, the antibody natalizumab reduces leukocyte entry across the BBB and is used in patients with relapsing-remitting MS. Charabati et al. [176] have identified dual immunoglobulin domain containing cell adhesion molecule (DICAM) as another potential target for impeding leukocyte entry into brain in MS. Epstein-Barr virus infection has been epidemiological linked to MS and Lanz et al. [177] found that a transcription factor (EBNA1) in that virus molecularly mimics glial cell adhesion molecule (GlialCAM).Interestingly, they found cross-reactive antibodies in CSF from MS patients. The gut-brain axis may also be important in MS. Ntranos et al. [178] have identified microbially derived metabolites of tryptophan and phenylalanine that can enter the brain and may induce neurotoxicity in MS.

Surprisingly, the lung also harbors a microbiome that appears to influence significantly the susceptibility for developing CNS autoimmune disease such as multiple sclerosis. Using selective antibiotics to manipulate the lung microbial population, Hosang et al. [179] demonstrated that the pulmonary microbiome regulates the immune reactivity of central nervous tissue and thereby influences its susceptibility to autoimmune disease development. Thus, a lung-brain axis may emerge as an important link between the periphery and CNS disease.

In an animal MS model (experimental autoimmune encephalitis), Hermans et al. [180] have identified a role of the oncostatin-M/CCL20 axis in BBB impairment and T helper 17 cell recruitment. While BBB dysfunction may be secondary to neuroinflammation, Nishihara et al. [181] found evidence of an intrinsic BBB impairment with MS when comparing iPSC-derived BMEC-like cells from control and MS patients. This suggests the brain endothelium is a therapeutic target.

In ALS, Yazdani et al. [82] have found that T cell phenotypes in blood and CSF are good predictors of disease progression with high CD4^+^FOXP3^−^ effector T cell expression being associated with poor survival, whereas a high frequency of Treg cells was associated with better survival. They also found evidence of clonally expanded CD4^+^ and CD8^+^ cells in CSF and propose that modulating adaptive immunity is a therapeutic option for ALS.

### Psychiatric disorders

There is growing awareness that altered BBB function may play an important role in psychiatric disorders such as depression [182]. For example, Dion-Albert et al. [183] have found that chronic social and sub-chronic variable stress promotes BBB alterations in female mice and that inducing localized BBB disruption in the prefrontal cortex induces anxiety- and depression-like behaviors. Importantly, they have identified soluble E-selectin (an adhesion molecule expressed on endothelial cells) in the serum of women with major depressive disorders. Matsuno et al. [184] have identified a role of vascular endothelial growth factor (VEGF) and its receptor VEGFR2 in stress-induced depression-like behavior in mice.

Dai et al. [185] have found an impairment in meningeal lymphatics in a mouse model of depression (sub-chronic variable stress) which only occurred in females. Manipulating the meningeal lymphatics altered this sex-difference in susceptibility to stress-induced depression- and anxiety-like behaviors. Together with the work at the BBB, this suggests that changes in brain extracellular composition (either due to greater influx at the BBB or reductions in clearance systems) may contribute to depression- and anxiety-like behaviors.

## Drug delivery

### Enhancing drug delivery across the blood-brain barriers

There continues to be great interest in the use of focused ultrasound with microbubbles to transiently open the BBB for drug delivery (see [186] for a recent review) and it is being used in clinical trials. For example, Epelbaum et al. [187] have developed an implantable ultrasound device that they have used to repeatedly disrupt the BBB in patients with mild Alzheimer’s disease (NCT03119961). There has been less interest in disrupting the blood-CSF barrier at the choroid plexus but Kung et al. [188] report that a single low energy shockwave pulse can transiently disrupt that barrier in rats.

As described earlier, increasing claudin-5 expression to reduce BBB permeability may be of benefit in a number of neurological diseases. By contrast, reducing claudin-5 expression/function may enhance drug delivery across the BBB. Wakayama et al. [189] review the current state of such research and potential safety concerns. While transient reductions in claudin-5 can enhance drug delivery, chronic reductions cause adverse effects in mice and non-human primates.

There has been research into using extracellular vesicles to carry cargo across the BBB although more in vivo work is required in mammals [190]. The choroid plexus epithelial cell is one source of extracellular vesicles and Pauwells et al. [191] found such vesicles home to the brain and choroid plexus when delivered systemically. The vesicles can deliver cargo to astrocytes and microglia. Marra et al. [113] have found that extracellular vesicles from a subset of endothelial progenitor cells can help protect the NVU in ischemic/neurodegenerative retinopathy.

Delivery of therapeutic agents to brain tumors remains challenging with the blood-brain tumor barrier being very heterogeneous (reviewed in [192]). A wide range of innovative techniques are being developed to deliver effective drug concentrations to tumors (e.g., [193, 194]).

For gene therapy, AAVs that specifically target the brain and cross the BBB would be of great use. Goertsen et al. [195] have described an AAV capsid variant that causes brain-wide transgene expression and has reduced delivery to liver after systemic administration. The variant was effective in mice and non-human primates. AAV delivery of hexosaminidase has been employed in a clinical trial for Tay-Sachs disease, although direct delivery to the CSF/brain was utilized [196]. Duan et al. [197] have used an AAV that penetrates the BBB to deliver Cas9 and a single-guide RNA to target a mutant APP allele in a mouse Alzheimer’s disease model. Brain Aβ deposition, microgliosis, neurite degeneration and cognitive impairment were ameliorated. Recently, Grashoff et al. [198] have used AAVs to deliver short regulatory DNA sequences to enhance tight junction and transporter expression at the brain endothelium.

### The blood-brain barriers as therapeutic targets

While most attention has focused on enhancing drug delivery across the blood-brain barriers, those barriers themselves may be therapeutic targets. Martin et al. [160] have targeted Wnt signaling at the BBB by genetically engineering Wnt7a ligands into BBB-specific Wnt activators to avoid pleiotropic effects. They found benefit in both glioblastoma and ischemic stroke models.

Jang et al. [199] found that the chemotherapeutic agent methotrexate impacted non-cancerous choroid plexus cells. A choroid plexus-targeted AAV was used in mice to increase expression of the antioxidant enzyme superoxide dismutase 3. This strategy appeared to increase the antioxidant defense capacity of CSF and reduce methotrexate-induced hippocampal lipid peroxidation and the associated learning and memory deficits.

## Conclusions

The Editors-in-Chief of *Fluids and Barriers of the CNS* thank the authors, reviewers and editorial board members for their contributions to advancing the field in the past year and their support for the journal.

## Data Availability

Not applicable.
